# An Integrated System for *Geobacillus* Protoplast Formation, Transfection, Recombinant Genomes Rebooting, Efficient Propagation, and Genomic DNA Isolation of the Thermophilic Bacteriophage TP‐84

**DOI:** 10.1002/mbo3.70421

**Published:** 2026-09-24

**Authors:** Ireneusz Sobolewski, Katarzyna Adamowicz, Jan Chodorski, Piotr M. Skowron, Agnieszka Zylicz‐Stachula

**Affiliations:** ^1^ Department of Molecular Biotechnology, Faculty of Chemistry University of Gdansk Gdansk Poland

**Keywords:** DNA purification, genomics, *Geobacillus stearothermophilus*, phage display, protoplasts, thermophile, TP‐84 bacteriophage

## Abstract

Protoplast transformation has been an effective method for introducing plasmid DNA into *Bacillus* species; however, its application to deliver complete bacteriophage genomes has not been demonstrated previously. Here, we describe an optimized and reproducible method for forming *Geobacillus stearothermophilus* protoplasts and efficiently transfecting them with purified recombinant or wild‐type genomic DNA from the thermophilic bacteriophage TP‐84. Systematic optimization of key parameters, including bacterial cell density, lysozyme concentration, polyethylene glycol type and concentration, and DNA input, enabled robust phage genomic DNA uptake and subsequent production of infectious phage particles. In addition, we optimized and scaled up a single‐layer solid‐state bacteriophage propagation procedure for integration into the TP‐84 genome‐engineering workflow, enabling the recovery of high‐titer lysates and sufficient genomic DNA for sequencing and subsequent genome manipulation from a single Petri dish. In our experimental system, solid‐state propagation also provided greater stability of recombinant phage variants than propagation in liquid bacterial cultures. Together, these methods establish an integrated protoplast‐based system for phage genomic DNA delivery, rebooting, propagation, and recovery, enabling controlled manipulation of bacteriophage genomes and facilitating the rapid generation and analysis of recombinant variants.

## Graphical abstract

The study presents an optimized, reproducible protoplast transfection method for *Geobacillus stearothermophilus*. The method enables efficient uptake of native and fully synthetic TP‐84 bacteriophage genomes, their “rebooting” in vivo and production of infectious phage particles. It also introduces a simplified, single‐layer solid‐agar propagation system that yields high‐titer phage lysates from a single Petri dish. This enables rapid and scalable generation and analysis of recombinant thermophilic phage variants. Created with BioRender.com/figure-j2gknzp (Żylicz‐Stachula 2026).

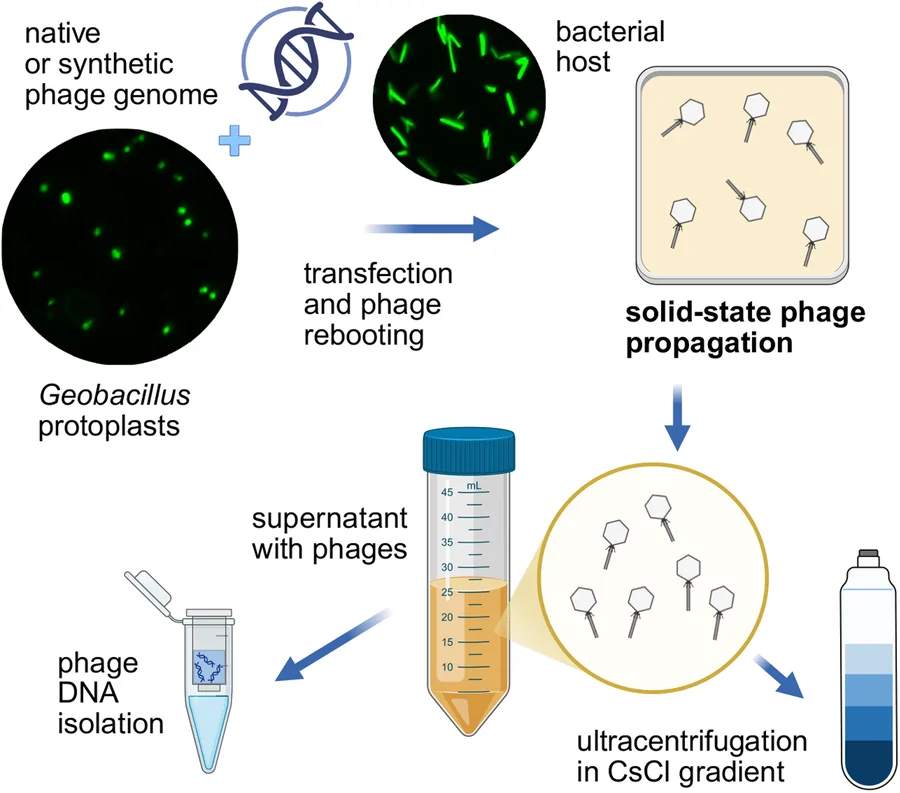

## 1 Introduction

Thermophilic bacteriophages are a special type of bacteriophage notable for their unusual molecular biology and potential applications in molecular biotechnology (Doss et al. 2023). Their ability to withstand a broad range of temperatures and harsh environmental conditions enables scientific studies of systems and environments inaccessible to standard model organisms. One example of such a thermophilic bacteriophage is TP‐84 (class Caudoviricetes, genus *Saundersvirus*, species *Saundersvirus* Tp84) (Kizer and Saunders 1972; Epstein and Campbell 1975), which was isolated in 1952 from greenhouse soil using *Geobacillus stearothermophilus* (*G. stearothermophilus*) as host (Saunders and Campbell 1966). The TP‐84 bacteriophage exhibits lytic activity against only a limited number of thermophilic *G. stearothermophilus* (Saunders and Campbell 1966) and *Parageobacillus* strains (historically classified as *Bacillus stearothermophilus*) (Welker 1988). These Gram‐positive, rod‐shaped bacteria can form heat‐resistant endospores and are commonly found in hot springs, compost, soil, and industrial environments, including dairy processing plants. *G. stearothermophilus* was initially identified and isolated from spoiled, canned corn in 1920 (Donk 1920). The TP‐84‐sensitive strains grow optimally at temperatures between 55°C and 65°C (Nazina et al. 2001); however, growth has been reported over broader temperature ranges, from 34°C to 69°C (Kakagianni et al. 2016), 40°C to 75°C (Kotzekidou 2014), or 31°C to 79°C (Łubkowska et al. 2023), depending on the strain and growth conditions.

TP‐84 is a bacteriophage whose genome organization and proteome have been extensively characterized by us (Skowron et al. 2024, 2018), establishing it as a versatile molecular platform with multiple potential applications. One such application is a novel thermostable phage display system we recently developed that exploits TP‐84 as a viral scaffold for protein engineering (Skowron et al. 2025). The TP‐84‐based phage display system can be used in scientific research, the pharmaceutical industry for the development and production of new vaccines and biological drugs, in environmental protection (plant protection products and pollutant elimination), and in other industrial applications (Harada et al. 2018).

From a technical perspective, to obtain a phage display system, it is necessary to modify the bacteriophage genome, introduce it into host cells, and obtain subsequent generations of the designed phage mutants. Several techniques are currently in use for engineering recombinant or fully synthetic (in our approach) phages: homologous recombination, bacteriophage recombineering of electroporated DNA, in vivo recombineering, CRISPR‐Cas‐mediated genome engineering, in vitro rebuilding/refactoring of phage genomes, whole‐genome synthesis and assembly from synthetic oligonucleotides, yeast‐based assembly of phage genomes, and cell‐free transcription‐translation systems (Pires et al. 2016). However, current phage‐editing techniques remain challenging and are effective for only a limited number of mesophilic phages and their host microorganisms. In particular, genetic modification of phages infecting Gram‐positive bacteria is difficult due to the structural organization and defense mechanisms of their host cells. The thick cell wall of Gram‐positive bacteria hampers the reintroduction of engineered viral genomes into recipient cells, thereby limiting efficient phage rebooting. One of the successful strategies to overcome these limitations is the use of L‐form bacteria—pleomorphic, cell‐wall‐deficient cells that retain metabolic activity and the ability to divide (Kilcher et al. 2018; Fernbach et al. 2024).

A fundamental requirement for cloning or constructing recombinant bacteriophages is the availability of readily transformable host cells. In Gram‐positive bacteria, three primary transformation methods are commonly used: electroporation (Dunny et al. 1991; Narumi et al. 1992), protoplast transformation (Imanaka et al. 1982; Chen et al. 1986), and triparental conjugation (Tominaga et al. 2016). Currently, each of these approaches involves time‐consuming preparatory steps, has low overall efficiency, and is associated with distinct advantages and limitations.

Protoplast transformation was one of the first effective methods for introducing exogenous DNA into Gram‐positive bacteria of the genus *Bacillus*. Classical studies on *Bacillus subtilis* demonstrated that protoplasts treated with polyethylene glycol (PEG) could efficiently take up circular plasmid DNA, leading to stable transformation and expression of the introduced genes (Chang and Cohen 1979). This method became the basis for further work on the genetic modification of other *Bacillus* species, including *B. licheniformis* and *B. amyloliquefaciens*, in which plasmid vector DNA was also primarily used, and the effectiveness depended on the host's ability to regenerate the cell wall and on the host's defense systems (Schaeffer et al. 1965; Gao et al. 2011). Protoplast transformation was subsequently adapted to thermophilic bacterial strains, historically classified as *B. stearothermophilus* and now included in the genera *Geobacillus* or *Parageobacillus* (Nazina et al. 2001; Coorevits et al. 2012; Aliyu et al. 2016, 2018). Imanaka et al. (1982) were the first to demonstrate the possibility of transforming *G. stearothermophilus* protoplasts using antibiotic‐resistance plasmids. Further work led to the development of a genetic system based on engineered *Parageobacillus* (historically *B. stearothermophilus*) strains NUB36/NUB3621 (Wu and Welker 1989). These studies primarily used shuttle plasmids capable of replication in *Escherichia coli* and *Geobacillus*, enabling their widespread use in the genetic engineering of thermophiles (Wu and Welker 1989; Vallier and Welker 1990). Significantly, the available literature on protoplast transformation in *Bacillus, Geobacillus*, and *Parageobacillus* has almost exclusively described the introduction of plasmid DNA or DNA fragments intended for homologous recombination. There are no reports documenting the routine or efficient introduction of purified bacteriophage genomic DNA into these bacteria via protoplast transformation. Studies on phages infecting *Bacillus* and *Geobacillus* have focused primarily on natural infection, transduction, or isolation of phages from lysates, rather than on the artificial introduction of complete phage genomes into protoplasts (Ackermann 2007; Calendar and Abedon 2005).

Here, we describe how to successfully use *G. stearothermophilus* 10 protoplasts as recipients of the TP‐84 bacteriophage genome (native or synthetic) to restart phage packaging. This approach provides a robust and suitable platform for rapid phage engineering. As we described elsewhere (Skowron et al. 2025), a fully functional modified TP‐84 phage genome can be designed in silico, synthesized, and successfully rebooted within the *Geobacillus* host within just a few days using our approach. As a proof of concept, we generated several recombinant virulent TP‐84 phages displaying specific markers on their capsids (Skowron et al. 2025).

## 2 Materials and Methods

### 2.1 Media and Reagents

The bacteriophage host was cultivated in TYM medium supplemented with streptomycin (Sigma‐Aldrich, St. Louis, MO, USA) at 50 µg/mL. The components of the TYM medium included: Peptone K (pancreatic casein hydrolysate) (BTL, Poland), yeast extract (BTL), MgCl_2_ (Sigma‐Aldrich, St. Louis, MO, USA), CaCl_2_ *x*H_2_O (Merck KGaA, Darmstadt, Germany), and fructose (Bioshop, Burlington, Canada). To precipitate the bacteriophage particles, PEG 8000 (Bioshop) was used. TP‐84 particles were then resuspended in TMC buffer (Tris‐MgSO_4_‐CaCl_2_ buffer), which consists of 10 mM Tris–HCl (Sigma‐Aldrich), 10 mM MgSO_4_ (Stanlab, Poland), and 5 mM CaCl_2_, pH 7.5. CsCl, used for the density gradient, was also sourced from Sigma‐Aldrich. For the protoplast transfection, PEG 4000 (Bioshop) was employed. The enzymes used during the DNA isolation procedure included RNase A (GeneON GmbH, Ludwigshafen, Germany), DNase I (A&A Biotechnology, Gdynia, Poland), and proteinase K (AppliChem Inc., Darmstadt, Germany). The polymerase chain reaction (PCR) mix used to confirm the presence of phages on questionable plates was obtained from A&A Biotechnology (Gdynia, Poland). DNA purification kits were sourced from New England Biolabs (Ipswich, MA, USA), while DNA standards were provided by ThermoFisher Scientific Baltics UAB (Vilnius, Lithuania). Synthesis of PCR primers was conducted by Eurofins Genomics (Germany GmbH). All the other chemicals were sourced from Sigma‐Aldrich. The recombinant DNA constructs, PCR fragments, and starters, as well as restriction endonuclease digestions, were designed using SnapGene software version 5.1.5 (http://www.snapgene.com).

### 2.2 Wild‐Type TP‐84 Bacteriophage Propagation, Purification, and Genomic DNA Isolation

Wild‐type bacteriophage TP‐84 was cultivated, and bacteriophage particles were purified by PEG/NaCl precipitation followed by CsCl density gradient ultracentrifugation, as previously described (Sobolewski et al. 2022), with a minor modification to increase recovery: the chloroform extraction step following PEG/NaCl precipitation was replaced by centrifugation at 10,000*g* for 10 min at room temperature. This modification reduced phage losses during purification, resulting in a higher phage yield in the final preparation. Bacteriophage quantification was conducted using the soft‐agar overlay technique (Saunders and Campbell 1966). The overlayer contained 3 mL of 0.65% agar in TYM broth; the base layer contained about 30 mL of 2% agar in the same broth (TYM agar). The plates were incubated overnight at 55°C before plaque counting.

Genomic DNA from wild‐type bacteriophage TP‐84 was isolated using a previously described protocol (Sobolewski et al. 2022), but with some modifications. The precipitated PEG/NaCl bacteriophage particles were resuspended in TMC buffer supplemented with DNase I and RNase A and incubated for 1 h at 37°C. Subsequently, sodium dodecyl sulfate (SDS) solution and proteinase K were added, and the suspension was incubated for 45 min at 60°C. Following protein digestion, one volume of binding buffer was added and mixed thoroughly. An equal volume of phenol‐chloroform mixture (pH 7.5) was then added, and the sample was shaken for 30 s. After centrifugation at 10,000*g* for 10 min at room temperature, the aqueous phase was recovered and applied to a silica minicolumn. DNA purification was subsequently performed as described previously (Sobolewski et al. 2022).

### 2.3 Protoplast Formation and Transfection Conditions Prior to Optimization

The initial protocol for transfection of *G. stearothermophilus* protoplasts with wild‐type TP‐84 phage genomic DNA was developed in our laboratory by adapting the plasmid DNA‐mediated *Geobacillus* protoplast transformation protocol of Blanchard et al. (2014). Streptomycin‐resistant derivative of *G. stearothermophilus* 10 (Gs10StrR) (Łubkowska et al. 2023) was used for protoplast preparation. Cultures were grown overnight to saturation in TYM medium (per liter: Peptone K, 20 g; yeast extract, 4 g; MgCl_2_, 10 mM; CaCl_2_, 5 mM; fructose, 0.5%), supplemented with streptomycin (50 µg/mL). Overnight cultures were diluted 1:50 and incubated at 55°C with shaking for 3–5 h until an OD_600_ of approximately 1.0 was reached. For each transfection, 2 mL of culture was harvested by centrifugation (5000*g*, 2 min, 10°C). Cell pellets were resuspended in 500 µL of osmotically stabilized protoplasting medium P (TYM containing 5% [w/v] fructose and 15 mM MgCl_2_, supplemented with streptomycin at 50 µg/mL). Lysozyme was added to a final amount of 20 µg (10 µL of a 2‐mg/mL solution), and the suspension was incubated for 20 min at 37°C. Conversion of cells into protoplasts was confirmed microscopically. Protoplasts were collected by centrifugation (1000*g*, 5 min, 10°C), washed twice with 900 µL of medium P, and finally resuspended in 200 µL of the same medium.

In all, 800 ng of wild‐type TP‐84 genomic DNA and 950 µL of freshly prepared PEG 8000 solution (prepared in medium P) were added to the washed protoplasts. The protoplast/DNA/PEG mixture was incubated for 5 min at 10°C, centrifuged at 1800*g* for 5 min at 10°C, resuspended in 300 µL of medium P, and incubated at 55°C with or without shaking. Subsequently, 200 µL of a fresh host culture (OD_600_ = 1.5) was added, and the suspension was incubated for 10 min at 55°C. The mixture was then combined with 3 mL of molten 0.65% agar prepared in TYM broth and poured onto agar plates using the soft‐agar overlay technique. Following overnight incubation at 55°C, phage plaques were enumerated.

A subsequently modified version of the initial procedure described above was used for the construction of the TP‐84 phage display system and was reported by Skowron et al. (2025). This previously published procedure was further systematically optimized in the present study.

### 2.4 Agarose Gel Electrophoresis and Analysis of Phage Genomic DNA

The concentration and purity of the isolated bacteriophage genomic DNA were determined spectrophotometrically based on the A260/A280 and A260/A230 ratios. DNA integrity was assessed by electrophoresis on a 1% (w/v) agarose gel in TAE buffer containing ethidium bromide and visualized using an Azure 600 Imaging System (Azure Biosystems, USA). DNA molecular size markers were included for reference.

### 2.5 Assessment of Infectious TP‐84 Phage Particle Contamination in Purified Genomic DNA Preparations

The presence of residual infectious TP‐84 phage particles in genomic DNA preparations was assessed using the soft‐agar overlay technique (Saunders and Campbell 1966). Briefly, approximately 10 μL, corresponding to one‐fifth of each purified DNA preparation, was mixed with 200 μL of a freshly prepared *G. stearothermophilus* 10 (StrR) culture and incubated for 10 min at 55°C. Subsequently, 3 mL of molten top agar was added, and the mixture was poured onto TYM‐based double‐layer agar plates. Following overnight incubation at 55°C, the plates were examined for plaque formation. The absence of plaques was interpreted as no detectable infectious TP‐84 phage particles in the tested DNA preparation.

## 3 Results and Discussion

### 3.1 Development of a Highly Efficient Protoplast‐Based Transfection Procedure

The protoplast transfection procedure was developed stepwise. An initial procedure, based on the method of Blanchard et al. (2014), was adapted for transfection with TP‐84 bacteriophage genomic DNA. A subsequently modified version was used to construct the TP‐84 phage display system, as reported by Skowron et al. (2025). In the present study, this previously published procedure was further systematically optimized, resulting in the final protocol described here.

Given the specific purpose of the procedure—to reboot infectious bacteriophage particles after uptake of phage genomic DNA by protoplasts—its efficiency cannot be assessed using the conventional measure used for bacterial transformation. Specifically, the procedure is not intended to generate bacterial transformants; therefore, transformation efficiency expressed as the number of transformants per microgram of DNA is not directly applicable to this system. Accordingly, the performance of the optimized procedure was evaluated based on the reproducible recovery of infectious phage particles and plaque formation following transfection.

**Figure 1:**
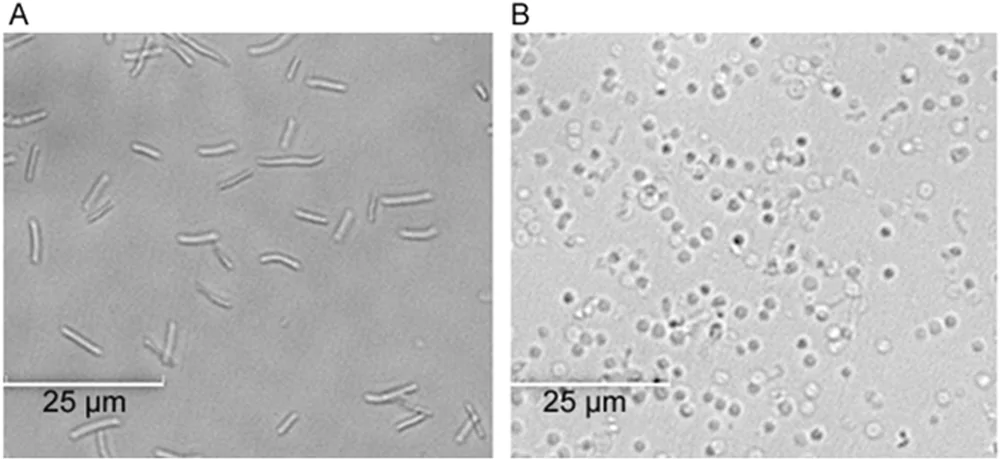
Bright‐field light micrographs acquired in transmitted light (TRANS) mode showing *Geobacillus stearothermophilus* 10 (StrR) (A) and protoplasts formed after lysozyme digestion (B). Photographs were taken using the CELENA S Digital Imaging System and objective 40X (Logos Biosystems).

Several parameters affecting the recovery of infectious TP‐84 phage particles following transfection of *G. stearothermophilus* 10 protoplasts with phage genomic DNA were systematically evaluated, including (i) the number of protoplast washing steps (Supporting Information Appendix, Figure A1), (ii) lysozyme concentration (0.02–0.2 mg/mL; Supporting Information Appendix, Figure A2), (iii) incubation time with lysozyme at 37°C (5–40 min; Supporting Information Appendix, Figure A3), (iv) the physiological state of the Gs10StrR culture (OD_600_ 0.6–1.8; Supporting Information Appendix, Figure A4), (v) the number of bacterial cells used for protoplast preparation (7.5 × 10^8^–3 × 10^9^ CFU; Supporting Information Appendix, Figure A5), (vi) the amount of genomic DNA (0.1–1.5 μg; Supporting Information Appendix, Figure A6), (vii) the volume of the DNA solution (5–40 μL; Supporting Information Appendix, Figure A7), and (viii) the molecular weight and concentration of PEG (PEG 4000, 6000, 8000, and 20,000 at concentrations ranging from 10% to 40%; Supporting Information Appendix, Figure A8). Protoplast formation was confirmed microscopically (Figure 1). Transfection performance was assessed using wild‐type TP‐84 genomic DNA as a model substrate.

The experiments conducted to optimize key parameters of the protoplast formation and TP‐84 DNA transfection protocol (Supporting Information Appendix, Figures A1– A8) led to the following conclusions:

1. Within the tested range, culture density (OD_600_) had no effect on the outcome when the same number of bacteria was used (Supporting Information Appendix, Figure A4). Therefore, the number of viable bacteria should always be determined by measuring the CFU/mL of the culture at a given OD_600_ prior to aliquoting. For protoplast preparation, the bacterial sample should contain no more than 7.5 × 10^8^–1.5 × 10^9^ CFU (Supporting Information Appendix, Figure A5), and lysozyme should be used at a final concentration of 0.02–0.08 mg/mL (Supporting Information Appendix, Figure A2). Increasing the amounts of bacteria or lysozyme greatly reduced transformation efficiency, thereby decreasing the number of viable phages obtained.
2. Two‐step washing of the protoplasts is essential to remove residual lysozyme. Omitting this step completely inhibited bacterial growth on agar plates (Supporting Information Appendix, Figure A1).
3. The highest efficiency of genomic DNA transformation into protoplasts was obtained using a 40% PEG 4000 solution (Supporting Information Appendix, Figure A8) and 0.2–1 µg of DNA (Supporting Information Appendix, Figure A6). The volume of DNA solution added should not exceed 10% of the protoplast suspension volume (Supporting Information Appendix, Figure A7).

The optimization experiments (Supporting Information Appendix, Figures A1– A8) identified conditions that markedly improved the protoplast transfection procedure previously developed by our group and reported by Skowron et al. (2025). Under optimized conditions, the yield of infectious TP‐84 phage particles increased approximately 130‐fold (Supporting Information Appendix, Figure A9) relative to that obtained using this earlier protocol.

**Figure 2:**
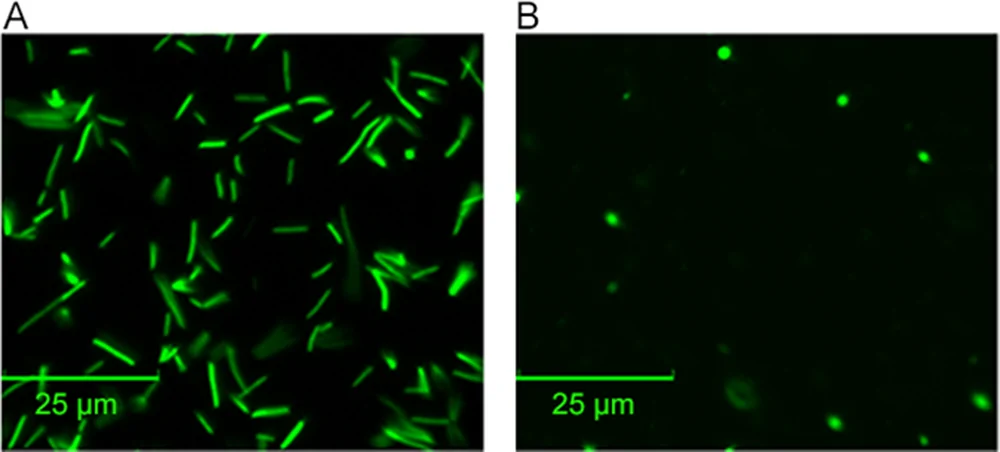
Fluorescence micrographs of *Parageobacillus genomosp*. 1 NUB3621 [pG1AK‐sfgfp] expressing the recombinant sfGFP gene (A) and its protoplasts formed after lysozyme digestion (B). Photographs were taken using the CELENA S Digital Imaging System and objective T40X (Logos Biosystems). The reduced number of cells visible in panel B reflects losses associated with protoplast preparation and handling; the image is intended to illustrate the morphological conversion of rod‐shaped *Parageobacillus* cells into spherical protoplasts.

Furthermore, the developed procedure was successfully applied to *Parageobacillus genomosp*. 1 NUB3621 strain (Figure 2), demonstrating its transferability beyond *G. stearothermophilus*. This finding supports the potential applicability of the procedure to other related bacterial hosts, although strain‐specific optimization may be required.

### 3.2 Recommended Protocol for *G. stearothermophilus* 10 Protoplast Formation and Transfection With TP‐84 DNA

The following protocol represents the final optimized procedure for protoplast formation and TP‐84 genomic DNA rebooting using *G. stearothermophilus* 10 (Gs10StrR), providing the highest and most reproducible recovery of infectious phage particles.

#### 3.2.1 Part I: Preparation of Host Bacteria Cell Pellets

1. Grow a fresh Gs10StrR culture to an OD_600_ of 0.6–1.5.
2. Harvest cells by centrifugation for 4 min at 5000*g* and 4°C.
3. Wash the pellet with cold TMC buffer using a volume equal to 1/5 of the original culture volume.
4. Aliquot the appropriate volumes of the suspension into 2‐mL Eppendorf tubes, centrifuge for 4 min at 5000*g* at 4°C, and completely remove the supernatant.
5. Freeze the resulting bacterial pellets at −80°C without adding a cryoprotectant and store until further use.
6. In the meantime, determine the viable cell concentration before freezing by preparing serial 10‐fold dilutions and plating at least two consecutive dilutions. Calculate the CFU/mL from plates within the reliable countable range, using multiple replicate plates for each selected dilution.
7. On the basis of the viable cell concentration (CFU/mL) determined before freezing, calculate the number of frozen cell pellet aliquots corresponding to 7.5 × 10^8^–1.5 × 10^9^ CFU.

#### 3.2.2 Part II: Protoplast Formation and Transfection

1. Thaw the required number of frozen cell pellet aliquots, corresponding to a total of 7.5 × 10^8^–1.5 × 10^9^ CFU as determined before freezing. Resuspend the thawed pellets in 1 mL of freshly prepared P medium supplemented with 0.08 mg/mL lysozyme and incubate for 30 min at 37°C to generate protoplasts.
2. Harvest the protoplasts by centrifugation (5 min at 1200*g*, 21°C), wash twice with 1 mL P medium, and resuspend the final pellet in 200 μL P medium.
3. Add 0.2–1.0 μg of bacteriophage DNA (in a solution volume not exceeding 15 μL), followed by 950 μL of freshly prepared 40% PEG 4000 in P medium. Mix gently and incubate for 5 min at room temperature.
4. Centrifuge the mixture (4 min at 1800*g*, 21°C), resuspend the pellet in 300 μL P medium, and incubate for 1 h at 55°C without shaking.
5. Add 200 μL fresh host culture (OD_600_ 1.5–2.0), incubate for 10 min at 55°C, then mix with 3 mL molten 0.65% agar in TYM broth and pour onto plates using the soft‐agar overlay method.
6. Incubate overnight at 55°C and evaluate transformation by the appearance of phage plaques.

### 3.3 Solid‐State Propagation of Bacteriophages in TYM Top Agar—Method Development and Validation

**Figure 3:**
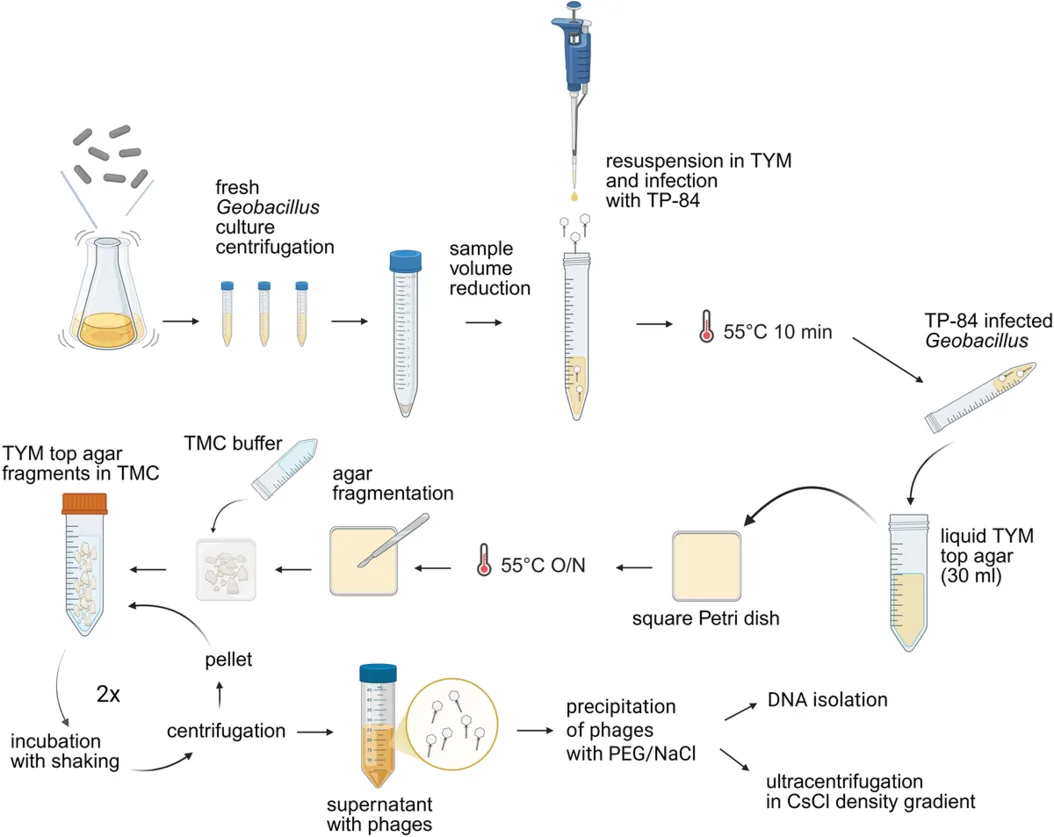
Scheme of solid‐state propagation of bacteriophages in TYM top agar. Created with BioRender.com/figure‐ri54zoy (Żylicz‐Stachula 2026). PEG, polyethylene glycol.

To facilitate efficient screening of new recombinant phage variants, we optimized a scaled‐up, single‐layer solid‐state propagation procedure that enables the recovery of sufficient phage material for genomic DNA isolation and subsequent sequencing (Figure 3).

Recovery of bacteriophages by washing or eluting plates exhibiting confluent or near‐confluent lysis is an established method for preparing plate lysates, including approaches employing a single agar layer without a bottom agar layer. Rizvi and Mora (1963) demonstrated plaque assays and confluent lysis on plates lacking bottom agar and reported phage yields comparable to, or slightly higher than, those obtained using conventional double‐layer plates (Rizvi and Mora 1963). More recent studies have also employed agar‐based phage recovery procedures (Manohar and Ramesh 2019; Tee et al. 2024).

Building on these established agar‐based propagation approaches, the present study focused on adapting and optimizing solid‐state propagation for its integration into a workflow designed for the rapid recovery and genomic analysis of engineered thermophilic TP‐84 variants. In our system, bacterial input, multiplicity of infection (MOI), agar volume, plate format, phage recovery, and subsequent genomic DNA isolation were experimentally optimized to enable phage amplification on a single 120 × 120 mm plate.

Application of the developed procedures to other bacteriophage‐host systems will likely require strain‐ and phage‐specific optimization. Protoplast formation depends on cell‐wall architecture, growth phase, lysozyme susceptibility, and osmotic stability, whereas DNA uptake may be influenced by PEG concentration, DNA size and topology, and host restriction or other defense systems. Following DNA uptake, successful phage rebooting also requires that the introduced genome remain biologically competent and initiate a complete viral replication and assembly program. Likewise, optimal solid‐state propagation parameters, particularly bacterial cell density, MOI, agar concentration, and incubation conditions, may differ among phages. Thus, the conditions established here should be regarded as an experimentally validated framework for TP‐84 that may provide a basis for adaptation to other phage‐host systems following appropriate optimization.

The following protocol represents the final optimized procedure for the solid‐state propagation of TP‐84 bacteriophages.

#### 3.3.1 Part III: Solid‐State TP‐84 Phage Propagation and Lysate Preparation Protocol

1. Dilute the overnight host culture 1/50 and incubate at 55°C for 3–5 h with shaking until OD_600_ reaches approximately 2.0, then harvest by centrifugation and resuspend the pellet in 4 mL fresh medium.
2. Infect the culture with TP‐84 lysate at an MOI of 0.3, incubate for 10 min at 55°C, mix with 30 mL of molten top agar, and pour into a square Petri dish.
3. Incubate overnight at 55°C until complete lysis is observed (transparent plate with no visible host).
4. Flood the plate with 25 mL of TMC buffer, disrupt the soft agar, and transfer the mixture into a centrifuge tube; shake briefly, then centrifuge at 12,000*g* for 30 min at 6°C.
5. Collect the supernatant, resuspend the remaining agar pellet in 25 mL TMC buffer, shake gently for 1 h, centrifuge again under the same conditions, and combine both supernatants to obtain the final TP‐84 lysate (approx. 60 mL).

Using the optimized procedure, the maximum number of bacteria that could be efficiently infected with TP‐84 phage at an MOI of 0.3 on a single square plate was approximately 1.5 × 10^10^ CFU. This corresponded to the bacterial pellet obtained from 35 mL of culture at an OD_600_ of 2.0, with a concentration of 4.05 × 10^8^ CFU/mL, as determined experimentally from the average colony counts of four plates inoculated with a 10^−6^ dilution. Under these conditions, approximately 60 mL of phage lysate with a titer of 5 × 10^10^ PFU/mL was recovered from a single plate, corresponding to a total yield of approximately 3 × 10^12^ PFU. The key parameters for achieving efficient propagation and complete bacterial lysis were the use of a concentrated host culture and an appropriate MOI (Supporting Information Appendix, Figure A10).

Overall, the optimized single‐layer solid‐state propagation procedure provided sufficient bacteriophage material for subsequent PEG 8000 precipitation, optional CsCl gradient purification, and isolation of microgram quantities of genomic DNA suitable for sequencing and further rounds of genome manipulation. The practical advantage of the procedure lies not in the use of a single agar layer per se, but in its integration as a downstream component of the TP‐84 genome‐engineering workflow, following protoplast‐based genome rebooting. The procedure enables the recovery of high‐titer phage material from a single plate using a relatively small volume of top agar. Moreover, in our experimental work, solid‐state propagation provided greater stability of recombinant TP‐84 variants than propagation in liquid bacterial cultures.

### 3.4 DNA Isolation after Solid‐State Phage Propagation

To fully exploit the high phage yields obtained with the optimized solid‐medium propagation procedure, a modified TP‐84 DNA isolation method was developed. The protocol involves PEG/NaCl precipitation of phage particles from agar‐extracted lysates, followed by enzymatic digestion of contaminating nucleic acids, protein removal, and silica‐column purification.

#### 3.4.1 Part IV: TP‐84 Genomic DNA Isolation Following Solid‐State Propagation

1. Precipitate phage particles from combined agar‐extraction supernatants by adding NaCl to a final concentration of 0.5 M and PEG 8000 to 5% (w/v). Incubate on ice for 2 h, then centrifuge for 40 min at 6000*g* at 4°C.
2. Resuspend the pellet thoroughly in 6 mL TMC buffer and centrifuge for 10 min at 8000*g* at 4°C to remove residual PEG. Resuspend the remaining pellet in 2 mL TMC buffer, centrifuge again under the same conditions, and combine both supernatants.
3. Add DNase I (0.1 U/mL) and RNase A (0.1 μg/mL) and incubate for 1 h at 37°C.
4. Add SDS to a final concentration of 0.1% (v/v) and Proteinase K to 0.25 mg/mL, then incubate for 45 min at 60°C.
5. Add two volumes of 5 M GuHCl/10 mM Tris solution, mix thoroughly, cool on ice, then add one volume of 96% ethanol. Mix and incubate for 30 min at 4°C.
6. Centrifuge for 40 min at 16,000*g* and 4°C, dry the DNA pellet, and resuspend it in 0.4 mL of 5 mM Tris–HCl (pH 7.5 at room temperature).
7. Add one volume of binding buffer and two volumes of chloroform. Mix by vortexing for 1 min, then centrifuge for 5 min at 10,000*g* at room temperature. Collect the aqueous phase and purify the DNA using a silica minicolumn as described by Sobolewski et al. (2022).

Depending on the titer of the starting lysate (agar‐extraction supernatant), the developed protocol routinely yielded 5–20 μg of purified TP‐84 genomic DNA, free of infectious phage particles. The amount and quality of DNA recovered were sufficient for subsequent applications, including DNA sequencing and the second round of protoplast transfection. Representative agarose gel electrophoresis profiles of genomic DNA preparations obtained from recombinant TP‐84 variants following solid‐state propagation are shown in Supporting Information Appendix, Figure A11.

## 4 Conclusions

1. This work demonstrates that the transformation of *Geobacillus* protoplasts can be used to directly introduce bacteriophage genomic DNA, significantly expanding the scope of this method. Previous reports of transformation of *Bacillus/Geobacillus* protoplasts have focused almost exclusively on circular plasmid DNA or DNA fragments targeted for homologous recombination, and there have been no descriptions of the routine and efficient introduction of complete phage genomes. Our results fill this gap, indicating that protoplasts can provide a functional platform for phage research independent of natural viral infection.
2. To construct a thermostable phage display system, we have developed an efficient technology for obtaining recombinant TP‐84 bacteriophages. In this method, we use our optimized procedure for introducing recombinant bacteriophage TP‐84 DNA into *G. stearothermophilus* protoplasts. After the introduction of DNA, recombinant bacteriophages are formed within protoplasts and can infect intact bacterial host cells. After the initial selection of potentially correct mutants by PCR and immunodetection, recombinant bacteriophages are efficiently amplified during cultivation of *G. stearothermophilu*s in a solid medium, purified, and used to isolate genomic DNA. As a result, we obtain microgram quantities of TP‐84 DNA from a single Petri dish. This allows for effective shortening of procedures, saving reagents, and testing many variants of bacteriophage genome modifications in a relatively short time. To date, we have obtained 30 stable recombinant bacteriophage variants through the TP‐84 genome engineering. These modifications included the introduction of a functional sfGFP‐encoding gene at multiple positions within the TP‐84 genome, as well as the engineering of selected TP‐84 genes by inserting DNA sequences encoding a His‐tag and an S‐tag (Skowron et al. 2025). The strategy we developed enables the generation of recombinant TP‐84 genome variants using both classical restriction enzyme‐based cloning and Gibson assembly.
3. Unlike approaches that rely on intact phage particles to deliver genetic material into bacterial cells, protoplast transfection enables the direct introduction of phage genomic DNA into host cells. Importantly, this approach enables the introduction of genetically modified phage genomes assembled in vitro, thereby facilitating the generation of recombinant phages and reducing the time required to obtain phage mutants. These features make protoplast transfection particularly useful for phage engineering and functional genomic studies. In this study, the procedure was successfully applied to *G. stearothermophilus* 10 and *Parageobacillus genomosp*. 1 NUB3621 strains. The developed procedure may also provide a basis for establishing protoplast‐based transfection of phage genomic DNA in other Gram‐positive phage‐host systems, although its application will likely require host‐ and phage‐specific optimization.
4. Compared with phage propagation in liquid bacterial cultures, solid‐state propagation demonstrated greater stability of recombinant phage variants, as indicated by our experimental observations, suggesting that this approach provides a more robust platform for maintaining genetic stability during phage propagation.

## Author Contributions

**Ireneusz Sobolewski:** conceptualization, investigation (lead), writing – original draft (lead), methodology, supervision. **Katarzyna Adamowicz:** investigation (supporting). **Jan Chodorski:** investigation (supporting), validation. **Piotr M Skowron:** funding acquisition, writing – review and editing (supporting), resources. **Agnieszka Zylicz‐Stachula:** writing – original draft (supporting), writing – review and editing (lead), resources, visualization.

## Ethics Statement

The authors have nothing to report.

## Conflicts of Interest

None declared.

## Supporting information


Supporting File

## Data Availability

All data generated or analyzed during this study are included in this published article.
