# Supplementary material for: An Integrated System for Geobacillus Protoplast Formation, Transfection, Recombinant Genomes Rebooting, Efficient Propagation, and Genomic DNA Isolation of the Thermophilic Bacteriophage TP‐84
Source: Microbiologyopen. 2026 Sep 24;15(5):e70421. doi: 10.1002/mbo3.70421 (PMC13602820; doi:10.1002/mbo3.70421)
Supplement: Supplementary file 1 [file MBO3-15-e70421-s001.pdf]

Appendix for

An integrated system for *Geobacillus* protoplast formation, transfection, recombinant genomes rebooting, efficient propagation and genomic DNA isolation of the thermophilic bacteriophage TP-84

Ireneusz Sobolewski*[1], Katarzyna Adamowicz[2], Jan Chodorski[3], Piotr M. Skowron[4] and Agnieszka Zylicz-Stachula[5]

[1]Department of Molecular Biotechnology, Faculty of Chemistry, University of Gdansk, Wita Stwosza 63, 80-308, Gdansk, Poland. ORCID: 0000-0002-9510-2557

*Corresponding author: ireneusz.sobolewski@ug.edu.pl; phone: +48 58 523 51 08

[2]Department of Molecular Biotechnology, Faculty of Chemistry, University of Gdansk, Wita Stwosza 63, 80-308, Gdansk, Poland; ORCID: 0000-0002-3972-5450; kachapoll@googlemail.com; phone: +48 691777126

[3]Department of Molecular Biotechnology, Faculty of Chemistry, University of Gdansk, Wita Stwosza 63, 80-308, Gdansk, Poland; ORCID: 0009-0005-6067-313X; jan.chodorski@phdstud.ug.edu.pl; +48 523 52 39

[4]Department of Molecular Biotechnology, Faculty of Chemistry, University of Gdansk, Wita Stwosza 63, 80-308, Gdansk, Poland; ORCID: 0000-0003-1345-3271; piotr.skowron@ug.edu.pl; phone: +48 58 523 52 42

[5]Department of Molecular Biotechnology, Faculty of Chemistry, University of Gdansk, Wita Stwosza 63, 80-308, Gdansk, Poland; ORCID: 0000-0003-2479-3634; agnieszka.zylicz-stachula@ug.edu.pl; phone: +48 58 523 51 08

An overnight *G. stearothermophilus* 10 (Str^R) culture was diluted 1:50 and grown for 4 h with shaking at 55°C until the cell density reached an OD_{600} of approximately 1.8. Aliquots of the culture were collected. The bacterial concentration was determined by calculating CFU/mL from the average number of colonies on four plates at a 10^{-6} dilution. An equal number of bacteria (1.5×10^9 CFU) was used in subsequent experiments.

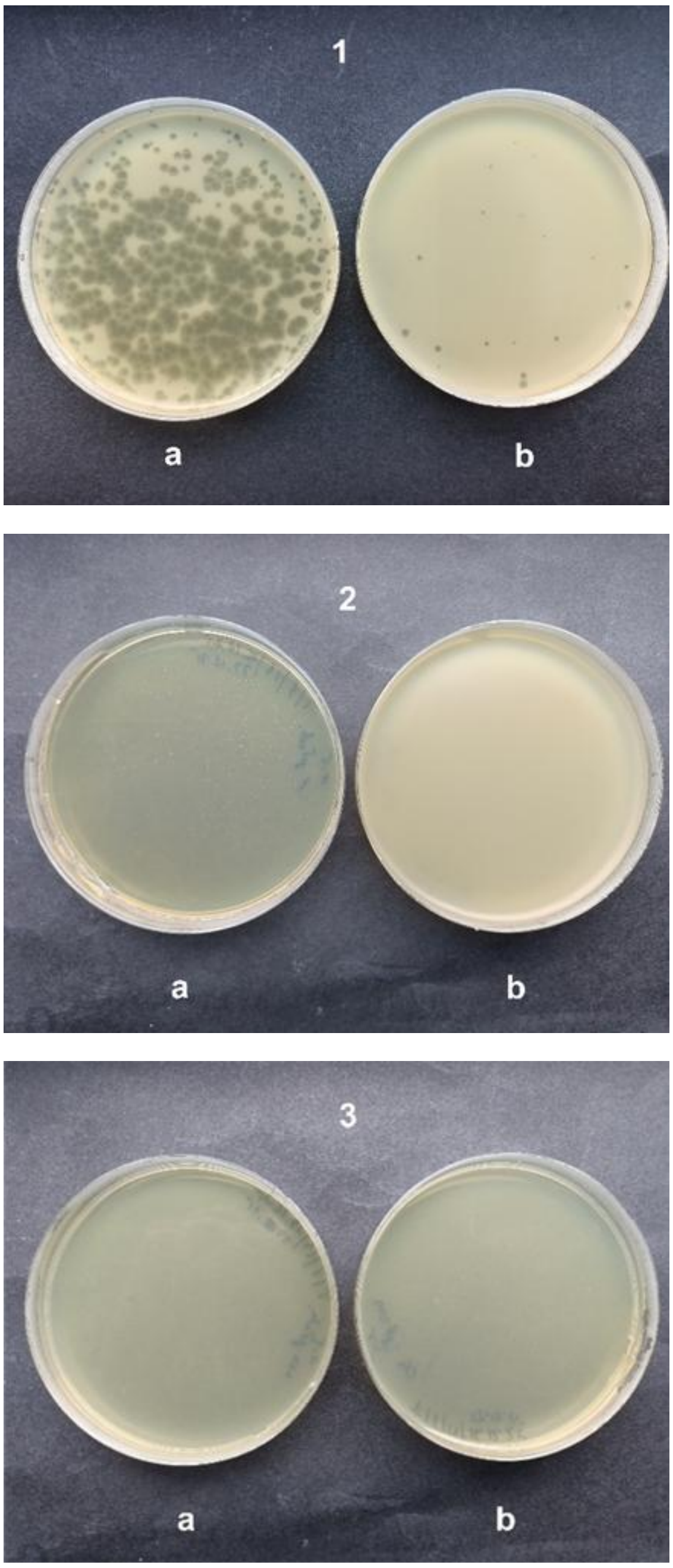

Fig. A1. Effect of the number of washing steps following lysozyme digestion on the recovery of infectious phage particles after protoplast transfection.

G. stearothermophilus 10 (Str^R) (1.5×10^9 CFU) was treated with lysozyme (0.08 mg/mL) in P medium and incubated for 20 min at 37°C to generate protoplasts. Following lysozyme treatment, the protoplasts were subjected to two, one, or no washing steps with P medium. The resulting protoplast preparations were subsequently transformed with 325 ng TP-84 genomic DNA according to the protoplast transformation procedure described by Skowron et al., 2025. Following transformation, 200 µL of a fresh *G. stearothermophilus* 10 (Str^R) host culture (OD_{600} = 1.5) was added to the transformed protoplasts. The mixtures were embedded in 0.6% TYM top agar, poured onto plates using the soft-agar overlay technique, and incubated at 55°C. Description of the photographs: 1, two washing steps; 2, one washing step; 3, no washing steps; a, 0.9 volume of the sample; b, 0.1 volume of the sample. The photograph shown in panel 1 (0.08 mg/mL lysozyme, two washing steps) is also presented in Fig. A2, panel 3, as this experimental condition was common to both optimization experiments.

An overnight *G. stearothermophilus* 10 (Str^R) culture was diluted 1:50 and grown for 4 h with shaking at 55°C until the cell density reached an OD_{600} of approximately 1.8. Aliquots of the culture were collected. The bacterial concentration was determined by calculating CFU/mL from the average number of colonies on four plates at a 10^{-6} dilution. An equal number of bacteria (1.5×10^9 CFU) was used in subsequent experiments.

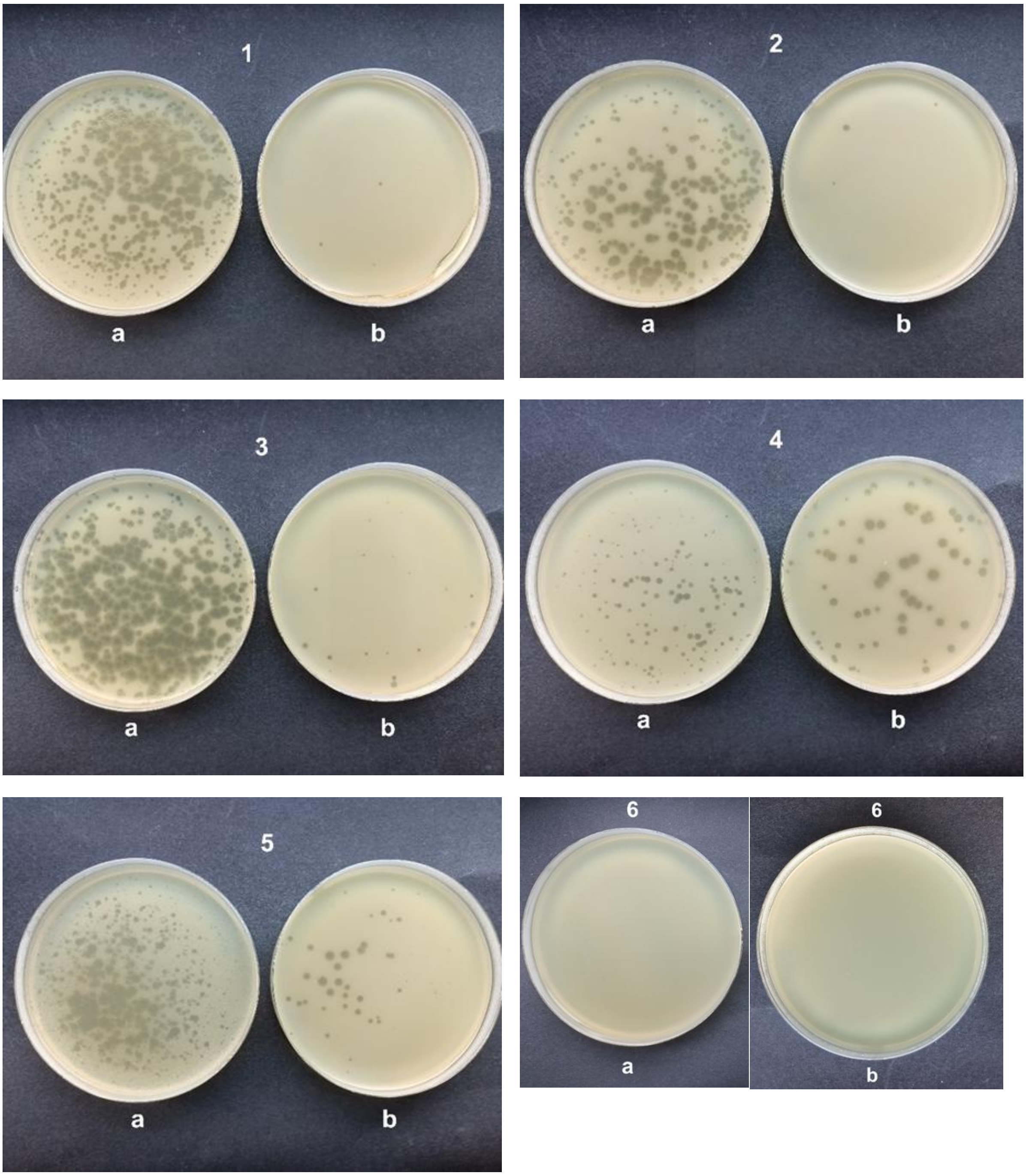

Fig. A2. Effect of lysozyme concentration on the recovery of infectious phage particles after protoplast transfection.

Protoplast preparation and transformation were performed as described for Fig. A1, using two washing steps after lysozyme treatment, except that different lysozyme concentrations (0.02 - 0.32 mg/mL) were tested. Description of the photographs: 1, 0.02 mg/mL lysozyme; 2, 0.04 mg/mL lysozyme; 3, 0.08 mg/mL lysozyme; 4, 0.16 mg/mL lysozyme; 5, 0.32 mg/mL lysozyme; a, 0.9 volume of the sample; b, 0.1 volume of the sample; 6, controls: a, DNA incubated with host; b, host culture. The photograph shown in panel 3 is also presented in Fig. A1, panel 1, as it represents the same experimental condition (0.08 mg/mL lysozyme and two washing steps).

Overnight culture was diluted 1/50 and propagated for 4 h with shaking at 55°C until the cell density reached an OD_{600} of approximately 1.37. Aliquots of the culture were taken, and the bacterial count was determined by calculating CFU/mL (average number of colonies from 3 plates with a culture dilution of 10^{-6}). The same number of bacteria (1.3×10^9 CFU) was used in the experiment.

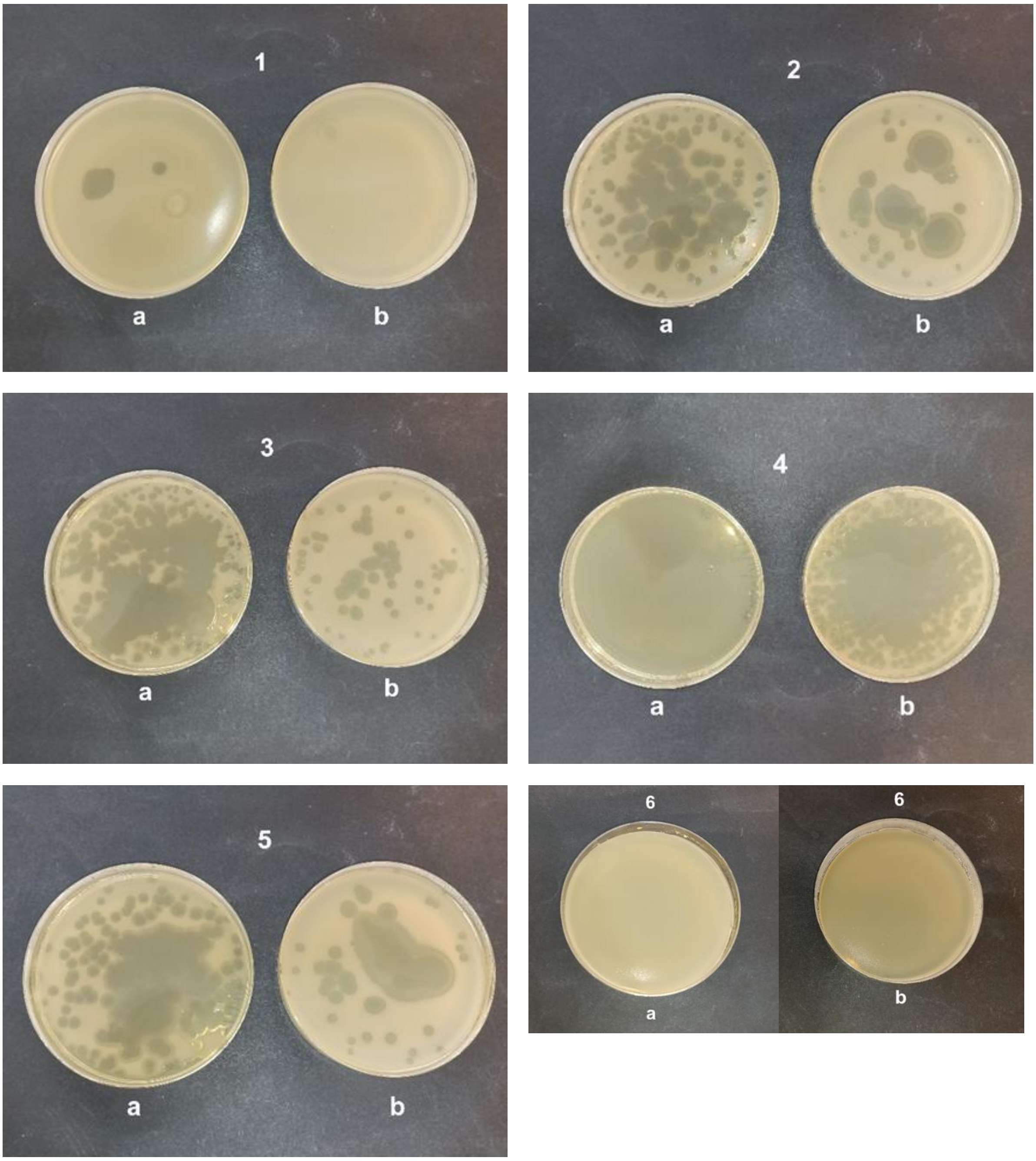

Fig. A3. Effect of lysozyme incubation time on protoplast formation and subsequent TP-84 genomic DNA transfection efficiency.

G. stearothermophilus 10 (Str^R) (1.3×10^9 CFU), 0.08 mg/mL lysozyme, and 300 ng of genomic DNA were used for protoplast preparation and transformation. To prepare protoplasts for transformation, bacteria were incubated with 0.08 mg/mL lysozyme for different time intervals. Resulted protoplasts were transformed with 300 ng of genomic DNA. Then, 200 µl of fresh host *G. stearothermophilus* 10 (Str^R) culture (OD_{600} = 1.5) was mixed with transformed protoplasts. The mixture was embedded in 0.6% agar TYM broth, poured onto plates using the soft-agar overlay technique, and incubated at 55°C. Description of the photographs: 1, 5 min at 37°C; 2, 10 min at 37°C; 3, 20 min at 37°C; 4, 30 min at 37°C; 5, 40 min at 37°C. a, 0.9 volume of sample; b, 0,1 volume of sample. 6, controls: a, DNA incubated with host; b, host culture.

An overnight *G. stearothermophilus* 10 (Str^R) culture was diluted 1:50 and grown for 4 h with shaking at 55°C until the cell density reached an OD_{600} of approximately 1.8. Aliquots of the culture were collected at OD_{600} values of 0.6, 1.0, 1.2, 1.5, and 1.8, centrifuged, and frozen. For each OD_{600} value, the bacterial concentration was determined by calculating CFU/mL from the average number of colonies on three plates at a 10^{-6} dilution. An equal number of bacteria (1.5×10^9 CFU) from each OD_{600} sample was used in subsequent experiments.

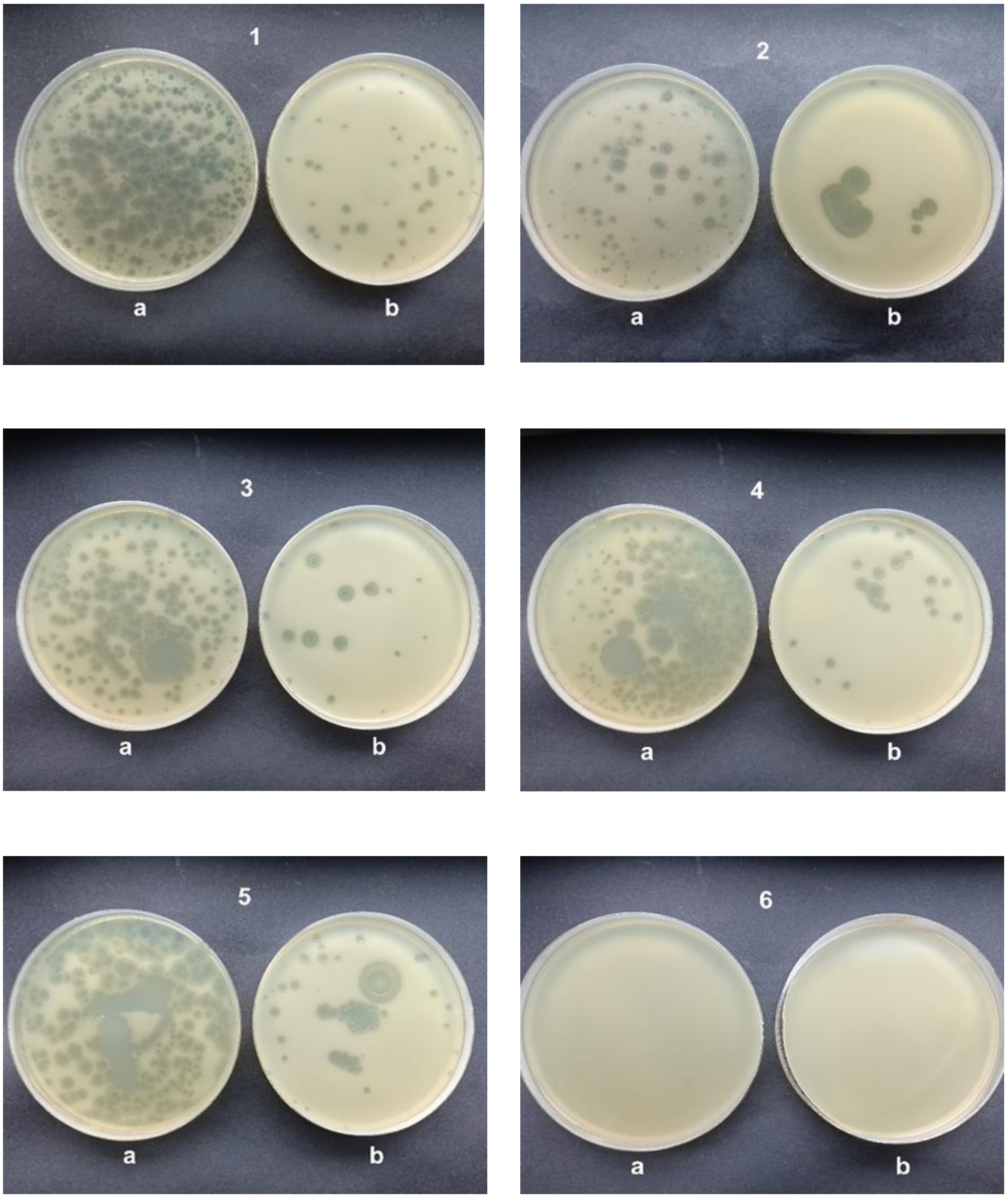

Fig. A4. The effect of cell density (OD_{600}) on protoplast transfection efficiency.

G. stearothermophilus 10 (Str^R) (1.5×10^9 CFU), 0.08 mg/mL lysozyme, and 325 ng of genomic DNA were used for protoplast preparation and transformation. Then, 200 µl of fresh host *G. stearothermophilus* 10 (Str^R) culture (OD_{600} = 1.5) was mixed with transformed protoplasts. The mixture was embedded in 0.6% agar TYM broth, poured onto plates using the soft-agar overlay technique, and incubated at 55°C. Description of the photographs: 1, OD_{600} 0.6; 2, OD_{600} 1.0; 3, OD_{600} 1.2; 4, OD_{600} 1.5; 5, OD_{600} 1.8; a, 0.9 volume of the sample; b, 0.1 volume of the sample. 6, controls: a, DNA incubated with host; b, host culture.

An overnight *G. stearothermophilus* 10 (Str^R) culture was diluted 1:50 and grown for 3 h with shaking at $55^{o}C$ until the cell density reached an OD_{600} of approximately 0.9. Aliquots of the culture were collected. The bacterial concentration was determined by calculating CFU/mL from the average number of colonies on four plates at a 10^{-6} dilution. Different numbers of bacteria (0.5 - 2.5 × 10^9 CFU) were used in the experiment. The lysozyme concentration was increased proportionally to the increase in bacterial numbers.

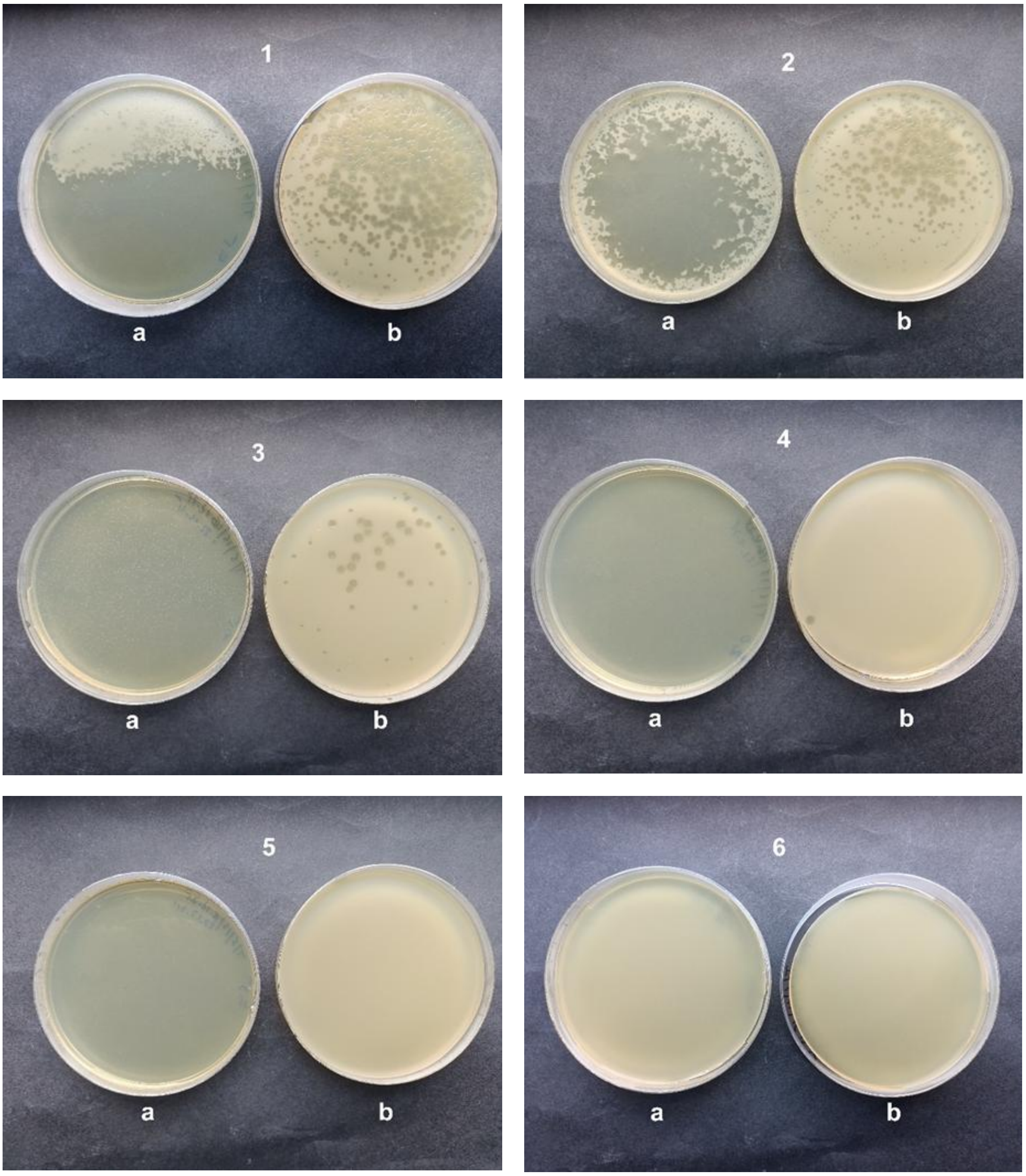

Fig. A5. Effect of bacterial cell number on protoplast transfection efficiency.

G. stearothermophilus 10 (Str^R) (0.5 – 2.5 x 10^9 CFU), 0.026 - 0.13 mg/mL lysozyme, and 325 ng of genomic DNA were used for protoplast preparation and transformation. Then, 200 µl of fresh host *G. stearothermophilus* 10 (Str^R) culture (OD_{600} = 1.5) was mixed with transformed protoplasts. The mixture was embedded in 0.6% agar TYM broth, poured onto plates using the soft-agar overlay technique, and incubated at $55^{o}C$. Description of the photographs: 1, 0.5 x 10^9 CFU / 0.026 mg/mL lysozyme; 2, 1.0 x 10^9 CFU / 0.053 mg/mL lysozyme; 3, 1.5 x 10^9 CFU / 0.08 mg/mL lysozyme; 4, 2.0 x 10^9 CFU / 0.1 mg/mL lysozyme; 5, 2.5 x 10^9 CFU / 0.13 mg/mL lysozyme; a, 0.9 volume of sample; b, 0,1 volume of sample. 6, controls: a, DNA incubated with host; b, host culture.

An overnight *G. stearothermophilus* 10 (Str^R) culture was diluted 1:50 and grown for 3 h with shaking at 55°C until the cell density reached an OD_{600} of approximately 0.9. Aliquots of the culture were collected. The bacterial concentration was determined by calculating CFU/mL from the average number of colonies on four plates at a 10^{-6} dilution. An equal number of bacteria (1.0×10^9 CFU) was used in subsequent experiments.

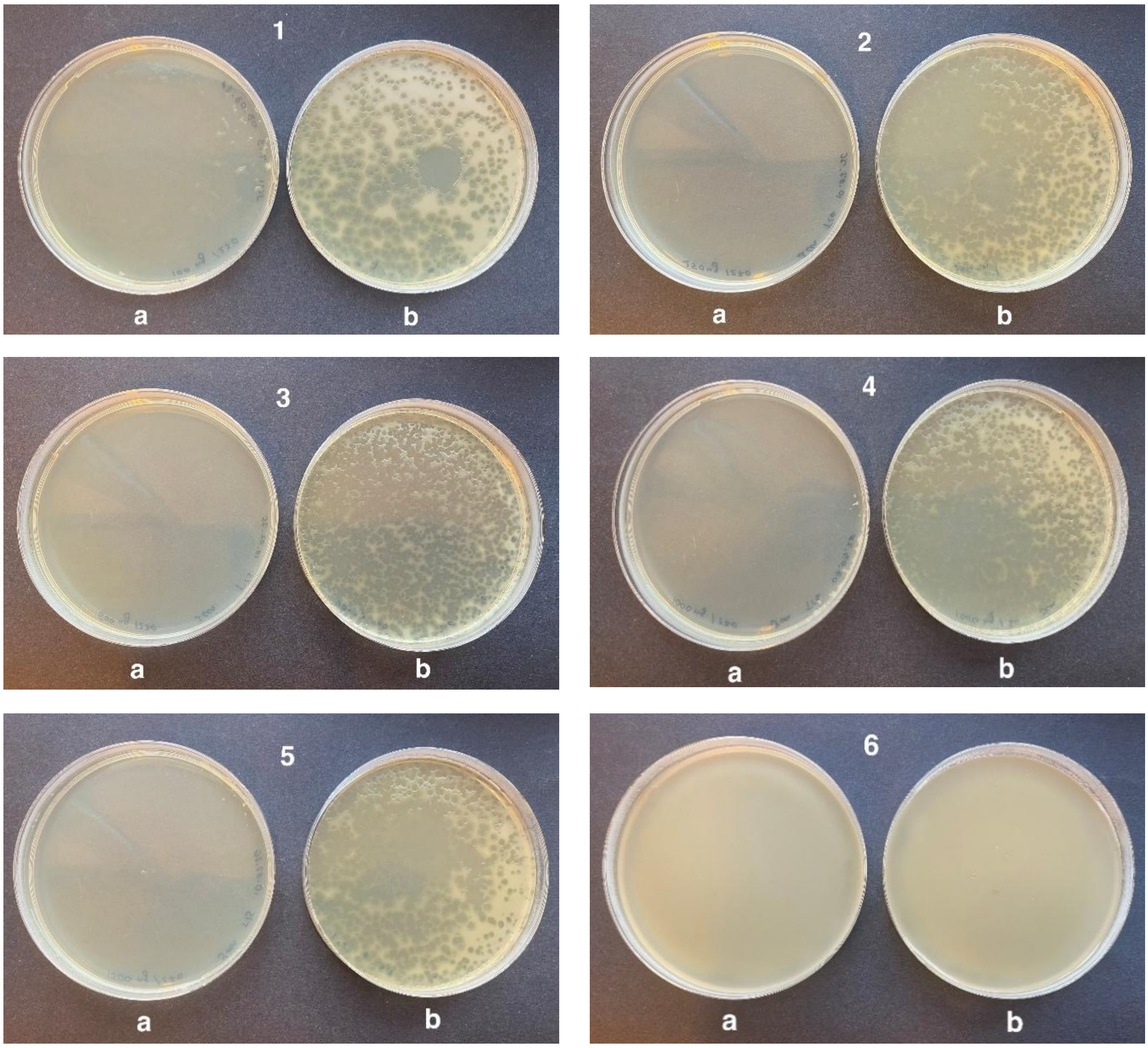

Fig. A6. Influence of DNA amount on protoplast transfection efficiency.

G. stearothermophilus 10 (Str^R) (1.0×10^9 CFU), 0.08 mg/mL lysozyme, and 0.1-1.5 µg of genomic DNA were used for protoplast preparation and transformation. Then, 200 µl of fresh host *G. stearothermophilus* 10 (Str^R) culture (OD_{600} = 1.5) was mixed with transformed protoplasts. The mixture was embedded in 0.6% agar TYM broth, poured onto plates using the soft-agar overlay technique, and incubated at 55°C. The following amounts of DNA resolved in 15 µL of 10 mM Tris-HCl buffer (pH 8.5 at 25°C) were used: 1, 0.1 µg; 2, 0.25 µg; 3, 0.5 µg; 4, 1.0 µg; 5, 1.5 µg; a, 0.9 volume of sample; b, 0.1 volume of sample. 6, controls: a, DNA incubated with host; b, host culture.

An overnight *G. stearothermophilus* 10 (Str^R) culture was diluted 1:50 and grown for 3 h with shaking at 55°C until the cell density reached an OD_{600} of approximately 0.9. Aliquots of the culture were collected. The bacterial concentration was determined by calculating CFU/mL from the average number of colonies on four plates at a 10^{-6} dilution. An equal number of bacteria (1.0×10^9 CFU) was used in subsequent experiments.

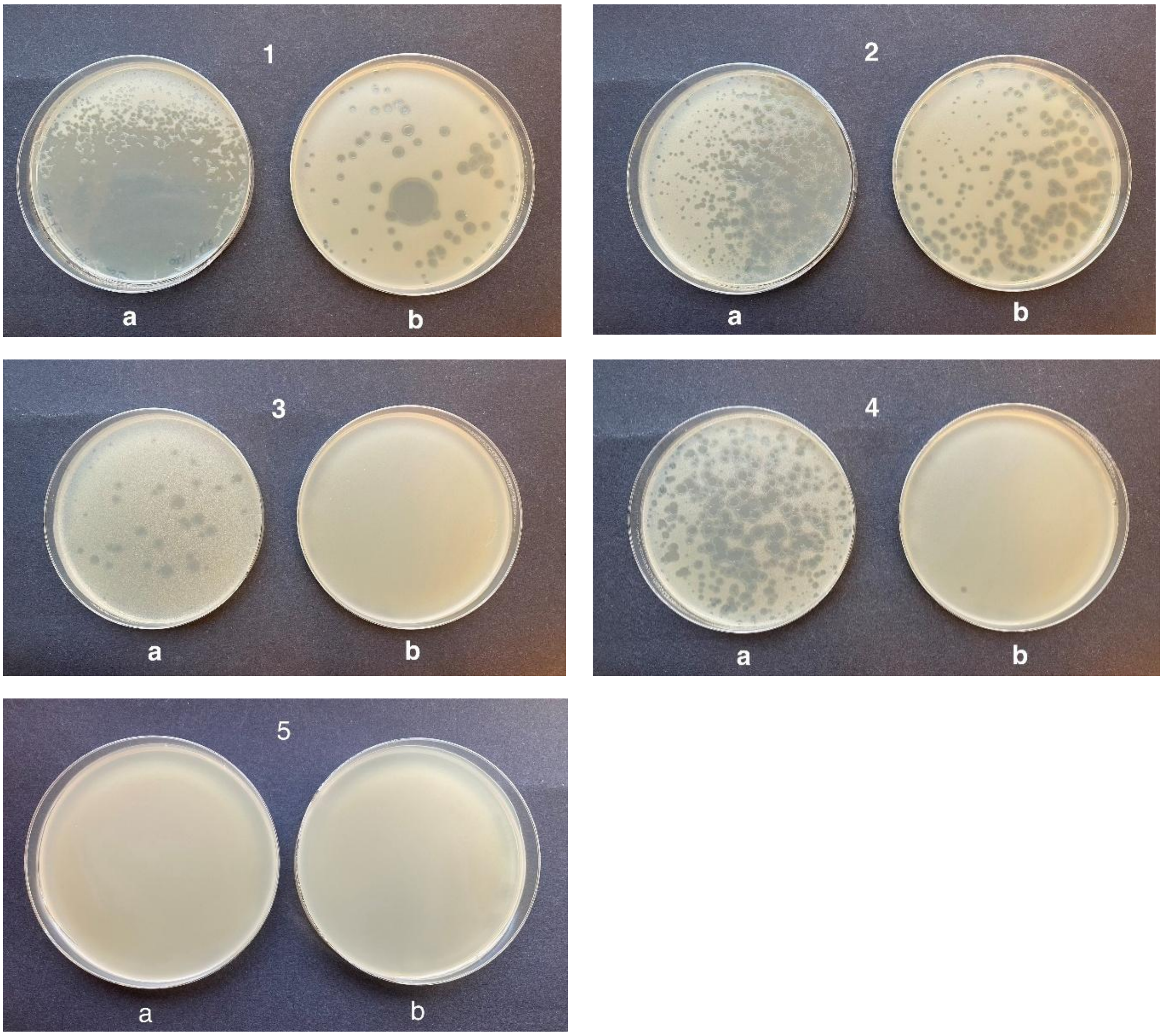

Figure A7. Influence of DNA solution volume on protoplast transfection efficiency.

G. stearothermophilus 10 (Str^R) (1.0×10^9 CFU), 0.08 mg/mL lysozyme, and 260 ng of genomic DNA dissolved in different volumes of 10 mM Tris-HCl buffer (pH 8.5 at 25°C) were used for protoplast preparation and transformation. Then, 200 µl of fresh host *G. stearothermophilus* 10 (Str^R) culture (OD_{600} = 1.5) was mixed with transformed protoplasts. The mixture was embedded in 0.6% agar TYM broth, poured onto plates using the soft-agar overlay technique, and incubated at 55°C. Description of the photographs: 1, 5 µl; 2, 10 µl; 3, 20 µl; 4, 40 µl; a, 0.9 volume of sample; b, 0.1 volume of sample. 5, controls: a, DNA incubated with host; b, host culture.

An overnight *G. stearothermophilus* 10 (Str^R) culture was diluted 1:50 and grown for 3 h with shaking at 55ºC until the cell density reached an OD_{600} of approximately 0.9. Aliquots of the culture were collected. The bacterial concentration was determined by calculating CFU/mL from the average number of colonies on four plates at a 10^{-6} dilution. An equal number of bacteria (1.5×10^9 CFU) was used in subsequent experiments.

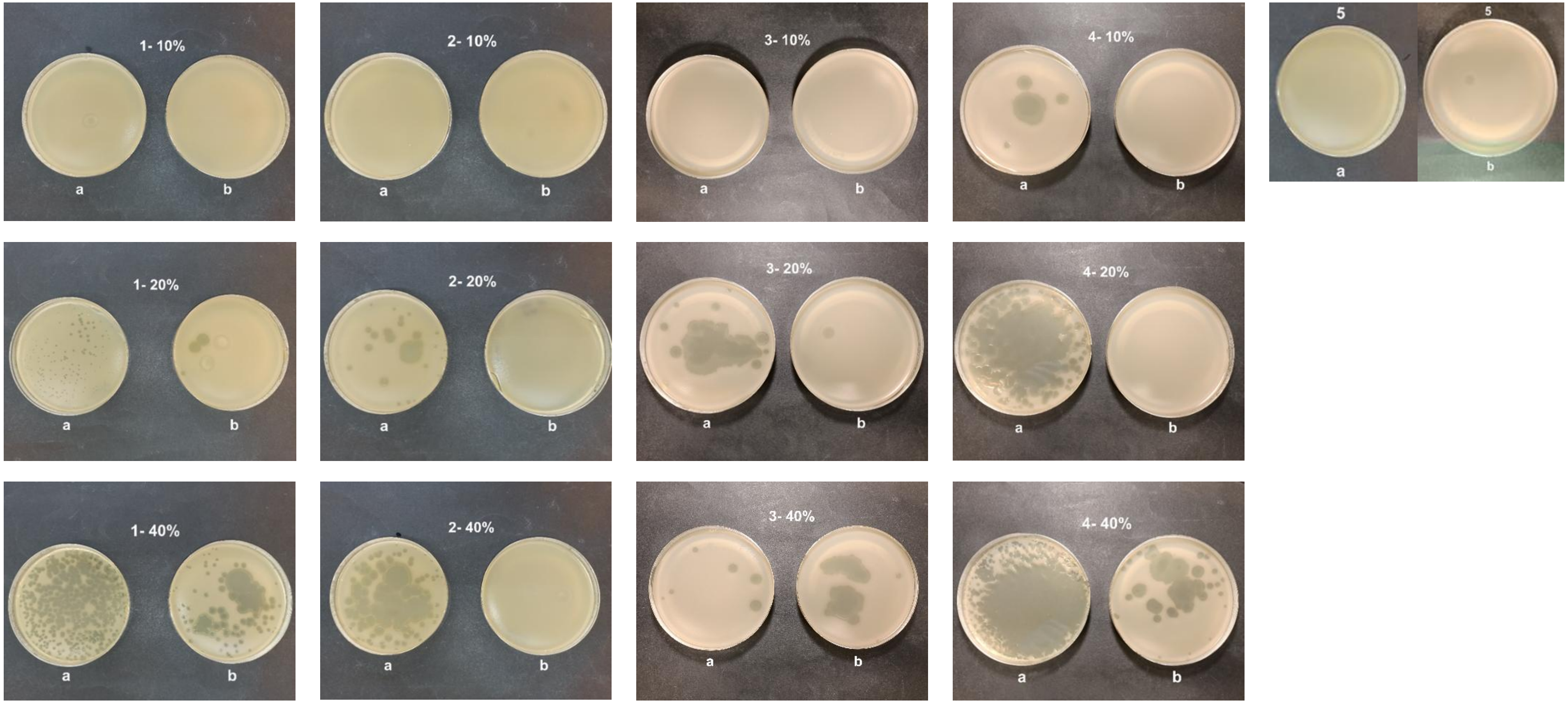

Figure A8. The effect of different PEGs on protoplast transfection.

G. stearothermophilus 10 (Str^R) (1.3×10^9 CFU), 0.08 mg/mL lysozyme, and 300 ng of genomic DNA were used for protoplast preparation and transformation. Then, 200 µl of fresh host *G. stearothermophilus* 10 (Str^R) culture (OD_{600} = 1.5) was mixed with transformed protoplasts. The mixture was embedded in 0.6% agar TYM broth, poured onto plates using the soft-agar overlay technique, and incubated at 55ºC. Description of the photographs: 1, solution of PEG4000: 10%, 20%, 40%; 2, solution of PEG6000: 10%, 20%, 40%; 3, solution of PEG8000: 10%, 20%, 40%; 4, solution of PEG20000: 10%, 20%, 40%; a, 0.9 volume of sample; b, 0.1 volume of sample. 5, controls: a, DNA incubated with host; b, host culture.

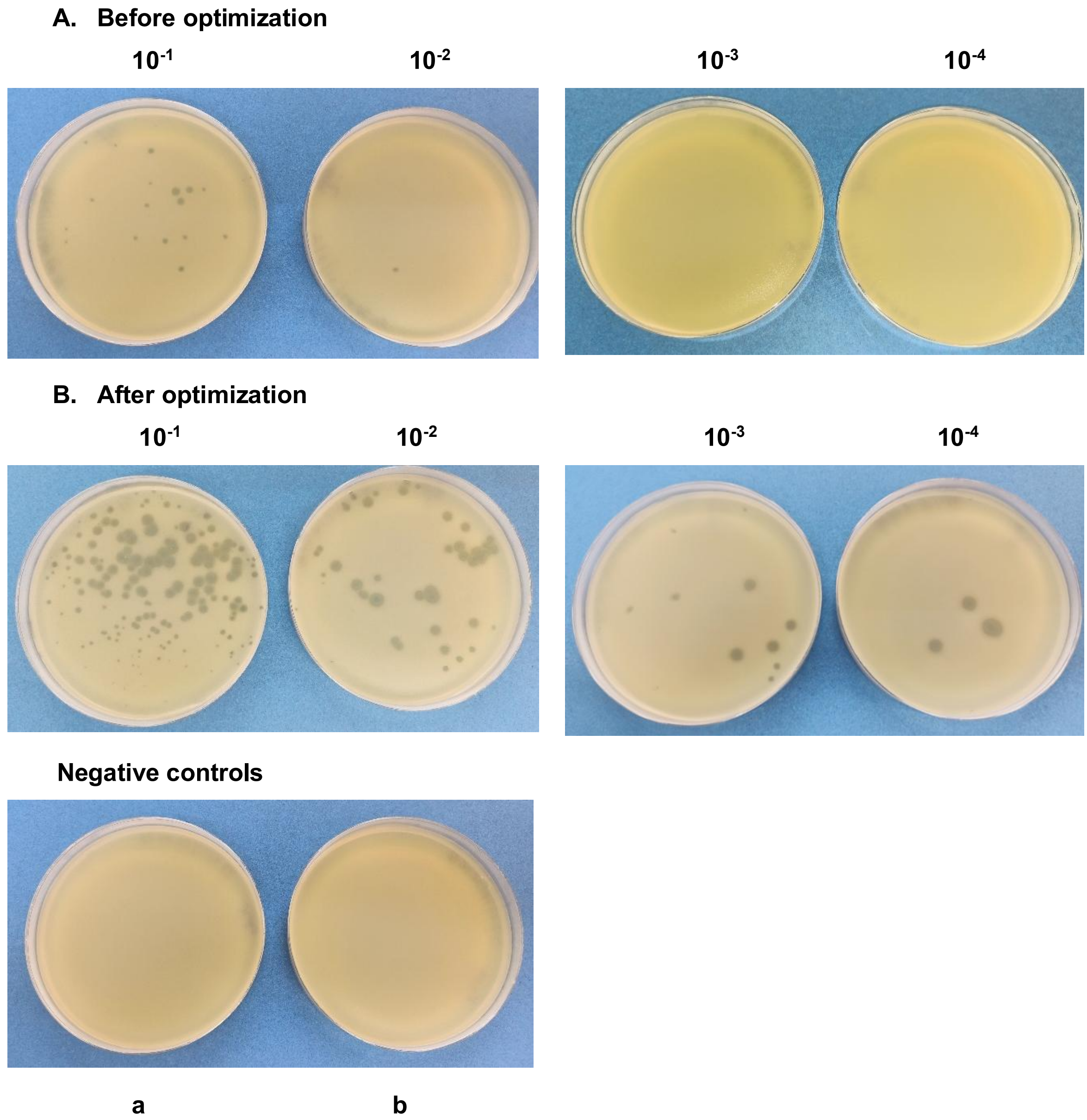

Fig. A9. Comparison of infectious TP-84 phage yield before and after transfection optimization.

Following transfection, serial tenfold dilutions of the protoplast suspension (10^{-1} - 10^{-4}) were mixed with fresh host cells, plated using the soft-agar overlay method, and incubated overnight at 55°C. The number of plaques was determined the following day, and the yield of infectious TP-84 phage particles was calculated as PFU/mL. The results obtained using the original and optimized transformation protocols were then compared. Negative controls included: (a) the bacterial host alone and (b) the bacterial host incubated with TP-84 genomic DNA.

(A) Protoplast formation and transfection were performed according to the transfection protocol described by Skowron et al., 2025. The transfection yielded infectious TP-84 phage particles with a titer of approximately 1.7×10^3 PFU/mL.

(B) Protoplast formation and transfection were performed according to the optimized transfection protocol (this work). Protoplasts were prepared from 1.5×10^9 CFU of *G. stearothermophilus* 10 (Str^R) using 0.08 mg/mL lysozyme and transformed with 250 ng of TP-84 genomic DNA, dissolved in 2 µL of 10 mM Tris-HCl (pH 8.5 at room temperature). The transfection yielded infectious TP-84 phage particles with a titer of approximately 2.2×10^5 PFU/mL.

Under the optimized conditions, the yield of infectious TP-84 phage particles increased approximately 130-fold compared with that obtained using the pre-optimization transfection protocol.

A frozen pellet prepared from a 50 mL culture at OD_{600} = 2.0 was resuspended in 100 mL of TYM medium and incubated with shaking at 55°C for 2 h, reaching an OD_{600} of 2.0. Aliquots were collected, and bacterial concentration was determined by calculating CFU/mL from the mean colony count of four plates at a 10^{-6} dilution. Two bacterial inputs, 1.33×10^{10} and 2.67×10^{10} CFU, were used. The bacterial culture was divided accordingly, centrifuged, and the pellets were resuspended in 4 mL of TYM medium. Infection with the recombinant TP-84 phage variant was performed at a titer of 5.1×10^{9} PFU/mL, resulting in a multiplicity of infection (MOI) of 0.38.

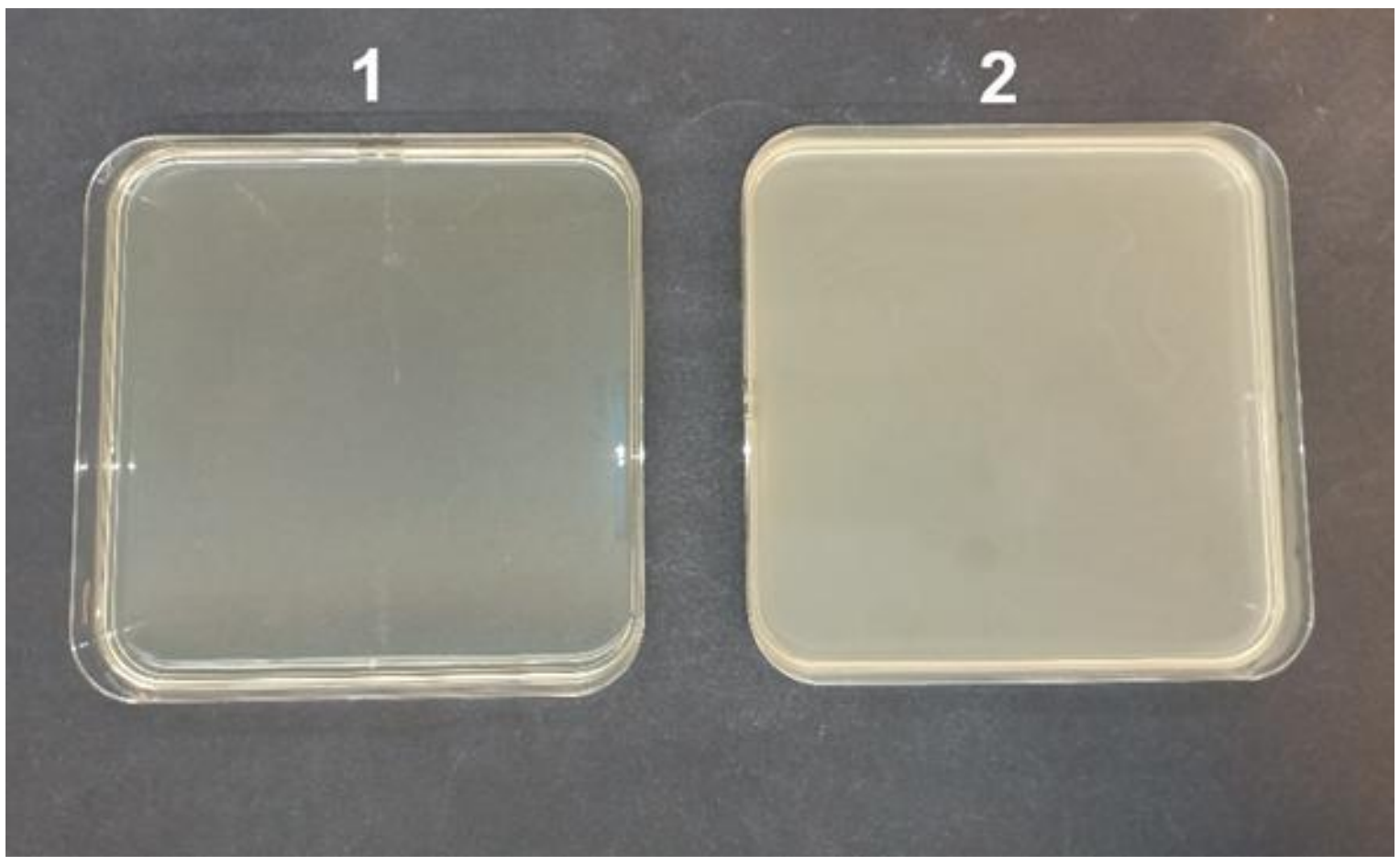

Fig. A10. Effect of bacterial cell input on TP-84 infection efficiency and host lysis during solid-state propagation.

The plate on the left shows complete lysis and transparency, whereas the plate on the right displays turbidity in the agar due to surviving bacteria. Concentrated *G. stearothermophilus* 10 (Str^R) were infected with the recombinant TP-84 phage variant at a titer of 5.1×10^{9} PFU/mL, embedded in 30 mL of 0.6% agar TYM medium, poured onto plates, and incubated at 55°C. Bacterial inputs were as follows: (1) 1.33×10^{10} CFU, and (2) 2.67×10^{10} CFU. Under these conditions, 60 mL of phage lysate with a titer of 5×10^{10} PFU/mL was obtained, corresponding to a total yield of 3×10^{12} PFU.

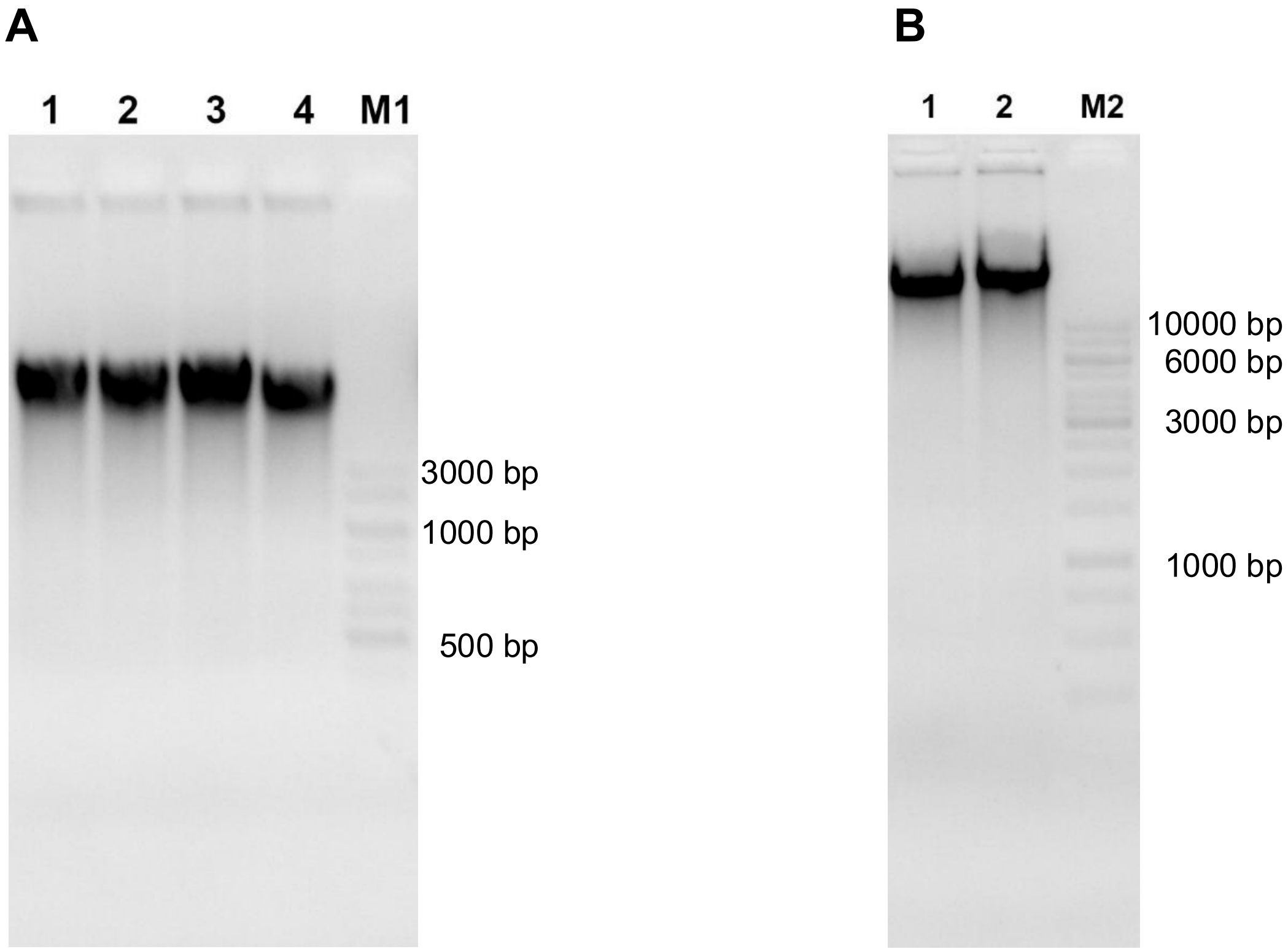

Fig. A11. Electrophoretic analysis of genomic DNA isolated from bacteriophages following solid-state propagation.

Genomic DNA samples were obtained during the screening of recombinant bacteriophage variants prior to sequencing. Solid-state bacteriophage propagation and genomic DNA purification were performed according to the procedure described in the Results section (this work). DNA samples were analyzed by electrophoresis on a 1% (w/v) agarose gel in TAE buffer and visualized as described in the Materials and Methods section (this work). Sample descriptions are provided in the table below. M1, GeneRuler™ 100 bp Plus DNA Ladder (Thermo Fisher Scientific); M2, GeneRuler™ 1 kb DNA Ladder (Thermo Fisher Scientific). Selected bands are indicated.

**Figure A10**	**Sample number**	**DNA concentration** [ng/µl]	**A260/A280**	**A260/A230**	**Total quantity** [µg]	**Amount applied to the well** [µl]
A	1	178	2.03	2.41	17.8	4
	2	181	2.03	2.28	18.1	4
	3	204	2.01	2.20	20.4	4
	4	178	2.02	2.21	17.8	4
B	1	162	1.98	2.59	16.2	4
	2	209	1.97	2.45	20.9	4

References

Skowron, P.M., Sobolewski, I., Adamowicz, K., Morcinek-Orłowska, J., Gaffke, L., Jaroszewicz, W., Łubkowska, B., Pierzynowska, K., Zylicz-Stachula, A., Węgrzyn, G., 2025. The first thermophilic phage display system. Mater Today Bio 33, 101960. https://doi.org/10.1016/j.mtbio.2025.101960
